# Pain characteristics and incidence of functional disability among community-dwelling older adults

**DOI:** 10.1371/journal.pone.0215467

**Published:** 2019-04-15

**Authors:** Keitaro Makino, Sangyoon Lee, Seongryu Bae, Songee Jung, Yohei Shinkai, Ippei Chiba, Hiroyuki Shimada

**Affiliations:** 1 Department of Preventive Gerontology, Center for Gerontology and Social Science, National Center for Geriatrics and Gerontology, Obu, Aichi, Japan; 2 Japan Society for the Promotion of Science, Tokyo, Japan; Appalachian State University, UNITED STATES

## Abstract

This study examined the association between pain characteristics and the incidence of functional disability among community-dwelling older adults. This prospective cohort study included 4,365 older adults (mean age 74.7 years, 53.5% female) living in community settings. Pain characteristics, including severity and duration of pain, were assessed in participants who also underwent monthly follow-up assessment of functional disability for 24 months based on the national long-term care insurance system. Among the 4,365 participants, 2,149 (48.7%) reported pain, regardless of severity and duration. Of the 2,149 participants with pain, 950 (44.2%) reported moderate to severe pain and 1,680 (78.2%) reported chronic pain. Based on the univariate analyses, participants with moderate (hazard ratio [95% confidence interval]: 1.48 [1.05–2.09]) or severe (2.84 [1.89–4.27]) pain and chronic pain (1.50 [1.15–1.95]) showed significantly higher risk of disability incidence than did those without pain. After adjusting for covariates, severe pain remained a significant predictor (hazard ratio [95% confidence interval]: 1.66 [1.05–2.62]), but moderate (1.00 [0.69–1.47]) and chronic pain (1.04 [0.77–1.40]) did not. Our results established that moderate to severe pain or chronic pain affects functional disability; in particular, severe pain was independently associated with the incidence of disability. Subjective complaints of pain do not always correspond to physical causes; however, simplified questions regarding pain characteristics could be useful predictors of functional disability in community-dwelling older people.

## Introduction

As society is aging, increasing international attention is being paid to disability-free life expectancy in addition to longevity. A systematic analysis of the Global Burden of Disease Study 2015 reported that among 310 diseases and injuries worldwide, lower back and neck pains have the highest number of years lived with disability, calculated by multiplying the prevalence of a disorder by the short- or long-term loss of health associated with that disorder [1]. Among community-dwelling older adults, the reported prevalence of any pain was over 50% [2,3,4,5]. It is known that older adults with daily pain are at higher risk of experiencing pain-related interference compared to younger adults [6]. Pain in older adults is related to adverse health outcomes such as physical and psychological deficits [7,8], physical inactivity [9], falls [10], depression [11], and dementia [12]. Therefore, pain may shorten the disability-free life expectancy of older people.

Several previous studies have reported that there is a relationship between pain and disability in activities of daily living among community-dwelling older adults using longitudinal data; however, their results were incongruent. Ojagbemi et al. reported that the prevalence of pain at baseline was associated with onset of functional disability within 5 years among community-dwelling older adults [13]. Kaiho et al. showed that moderate or severe pain was associated with functional disability after adjusting for covariates in a 5.7-year longitudinal study [2]. In contrast, Andrews et al. showed that older adults with moderate or severe pain were more likely to develop activities of daily living disability, but this relationship was not significant after adjustment for confounders in a 10-year longitudinal study [14]. Eggermont et al. also reported that pain severity was not associated with the onset of disability after adjustment for covariates in an 18-month longitudinal study [15]. From these studies, it may be concluded that subjective pain is associated with future disability; however, the level of pain associated with disability risk remains unclear.

Accordingly, we sought to examine the incidence rate of functional disability based on pain characteristics (severity and duration) among community-dwelling older adults in a 24-month prospective cohort study.

## Methods

### Participants

This prospective study involved community-dwelling older adults enrolled in a sub cohort of the National Center for Geriatrics and Gerontology–Study of Geriatric Syndromes. A total of 5,781 individuals underwent our baseline assessment. Inclusion criteria required that the participants were aged 65 years or older and lived in Nagoya or Obu city, Japan, at the time of examination (from June 2013 to September 2013).

This study included participants who completed baseline assessments of pain characteristics and follow-up assessments of disability based on the national long-term care insurance (LTCI) system. All assessments were carried out by well-trained nurses and study assistants at the community center. All staff received training from the authors on the protocols for administering the assessments before the study began. We excluded participants based on the following criteria: 1) presence of disability based on the LTCI system, at baseline (n = 51), 2) history of depression or dementia (n = 238), 3) Mini Mental State Examination (MMSE) score below 24 (n = 965), 4) missing data for pain characteristics or the above-described variables (n = 107), 5) death or relocation to another city during the follow-up period (n = 55). After exclusion, data from 4,365 participants were available for analysis in the present study.

Informed consent was obtained from all participants prior to their inclusion in the study, and the ethics committee of the National Center for Geriatrics and Gerontology approved the study protocol.

### Variables

#### Functional disability

Participants were followed monthly for incident certification for personal support or care in the LTCI system during the 24-month period. Every Japanese national aged 65 years or older is eligible for benefits (institutional and community-based services, but not cash) based strictly on functional (physical and mental) disability. The nationally uniform criteria for long-term care need certification were objectively established by the Japanese government, and the computer-aided standardized needs-assessment system categorizes people into seven levels of need [16]. The process of certification of personal support or care in the LTCI system is as follows: 1) an elderly person or caregiver contacts the municipal government to request official certification of their care needs; 2) a trained local government official visits the individual’s home to evaluate support or need for nursing care based on current physical and mental status; 3) after completion of the assessment, the results are inputted into a computer to calculate the standardized scores for physical and mental status and estimated time required for nine categories of care (grooming, bathing, eating, toileting, transferring, assistance with instrumental activities of daily living, behavioral problems, rehabilitation, and medical services), and a care-need level based on the total estimated time for care is assigned; 4) the care needs certification board, which includes physicians, nurses, and other experts of health and social services, reviews the data; and 5) the applicant is assigned the level of care required (certified support-level ranging from 1 to 2 or care-level ranging from 1 to 5). The eligibility of the individual receiving care via the LTCI system is reevaluated every 6 months. In the present study, the incidence of functional disability was defined as a new certification of the LTCI service at any level.

#### Pain characteristics

Pain characteristics including severity and duration of pain were assessed in face-to-face interviews by well-trained study assistants. Pain severity was measured using the following three-point verbal rating scale [17]: ‘mild pain,’ ‘moderate pain,’ and ‘severe pain.’ Pain duration was assessed verbally; pain persisting for at least 2 months [18,19] was defined as chronic pain and pain persisting for less than 2 months was defined as non-chronic pain in this study. If pain was present in multiple sites, data for the pain with the maximum severity or longest duration were obtained.

#### Other covariates

As covariates, sociodemographic variables including age, sex, number of medication use, and medical history of chronic diseases (hypertension, diabetes mellitus, heart disease, e.g., angina pectoris, myocardial infarction, and aortic aneurysm; osteoarthritis, and spinal diseases) were assessed by face-to-face interviews conducted by well-trained nurses. Medication use was assessed as the number of all drugs continuously prescribed by a doctor. The use of three or more medications on a daily basis was defined as polypharmacy [20]. Gait speed, global cognitive function measured by the MMSE, and depressive symptoms were included as covariates, and these assessments were conducted by well-trained study assistants. Gait speed was measured at a comfortable pace along a 6.4-m walkway. Data were collected from a 2.4-m active stretch in the middle of the walkway, and the mean gait speed of five trials was calculated; slow gait speed was defined as that under 1.0 m/s [21]. Depressive symptoms were assessed using the 15-item Geriatric Depression Scale (GDS) [22]. The participants who scored ≥ 5 out of 15 points on the GDS were considered to have depressive symptoms [22].

### Statistical analysis

Baseline characteristics were compared between participants who developed disability and those who remained independent using the Student’s t-test for continuous variables and the chi-squared test for categorical variables.

We calculated the cumulative incidence of functional disability during the follow-up period according to baseline pain characteristics using Kaplan-Meier curves. Intergroup differences were estimated by the log-rank test. Cox proportional hazards regression models were used to analyze associations between pain characteristics and incidence of functional disability after adjusting for covariates (age, sex, medical conditions including hypertension, diabetes mellitus, heart disease, osteoarthritis, and spinal diseases; polypharmacy, slow gait speed, and depressive symptoms).

All analyses were performed using IBM SPSS Statistics 22 (IBM Japan, Tokyo, Japan). The level of statistical significance was set at *P* < 0.05.

## Results

### Prevalence of pain, incidence rate of disability, and participant characteristics

Of the 4,365 participants who were enrolled in our study, 2,127 (48.7%) reported pain, regardless of severity and duration. In terms of pain severity, of the 4,365 participants, 1,190 (27.3%) reported mild pain, 697 (16.0%) reported moderate pain, and 240 (5.5%) reported severe pain at baseline. In terms of pain duration, of the 4,365 participants, 464 (10.6%) reported non-chronic pain (< 2 months) and 1,663 (38.1%) reported chronic pain (≥ 2 months) at baseline.

During the 24-month follow-up period, 239 (5.5%) participants developed disability. Compared with those who remained independent, participants who developed disability showed significantly older age (*P* < 0.001), significantly higher proportion of chronic diseases including hypertension (*P* = 0.003), diabetes mellitus (*P* = 0.020), cardiovascular diseases (*P* = 0.003), and osteoarthritis (*P* < 0.001); and significantly more prescribed medications (*P* < 0.001). Those who developed disability showed slower gait speed (*P* < 0.001), lower MMSE score (*P* = 0.002), and higher GDS score (*P* < 0.001). In terms of pain characteristics, those who developed disability showed significantly higher proportion of severe pain (*P* < 0.001) or significantly more persistent pain (*P* = 0.010; Table 1).

**Table 1:** Baseline characteristics of participants.

		Overall (*n* = 4,365)	Independent (*n* = 4,126)	Incident disability (*n* = 239)	*Test* *statistic*	*df*	*P*
Age ^a^	(years)	74.7 ± 5.0	74.4 ± 4.9	78.7 ± 5.3	12.143	261.697	< 0.001
Sex ^b^	(female, %)	2,335 (53.5)	2,193 (53.2)	142 (59.4)	3.563	1	0.059
Medical condition	(n, %)						
	Hypertension ^b^	2,036 (46.6)	1,902 (46.1)	134 (56.1)	8.980	1	0.003
	Diabetes mellitus ^b^	539 (12.3)	498 (12.1)	41 (17.2)	5.375	1	0.020
	Heart disease ^b^	771 (17.7)	712 (17.3)	59 (24.8)	8.688	1	0.003
	Osteoarthritis ^b^	919 (21.1)	845 (20.6)	74 (31.2)	15.302	1	< 0.001
	Spinal disease ^b^	941 (21.6)	880 (21.4)	61 (25.7)	2.442	1	0.118
Medication ^a^	(n/day)	3.2 ± 2.8	3.1 ± 2.7	4.6 ± 3.4	6.476	254.638	< 0.001
Gait speed ^a^	(m/sec)	1.18 ± 0.21	1.19 ± 0.20	1.01 ± 0.24	10.994	252.891	< 0.001
MMSE ^a^	(score)	26.9 ± 1.8	26.9 ± 1.7	26.5 ± 1.8	3.040	4363	0.002
GDS ^a^	(score)	2.8 ± 2.7	2.7 ± 2.6	3.8 ± 3.1	5.266	257.168	< 0.001
Pain ^b^	(n, %)	2,127 (48.7)	1,991 (48.3)	136 (56.9)	6.764	1	0.009
Pain severity^b^	(n, %)				28.958	3	< 0.001
	Mild	1,190 (27.3)	1,131 (27.4)	59 (24.7)			
	Moderate	697 (16.0)	650 (15.8)	47 (19.7)			
	Severe	240 (5.5)	210 (5.1)	30 (13.1)			
Pain duration ^b^	(n, %)				9.132	2	0.010
	Non-chronic	464 (10.6)	441 (10.7)	23 (9.6)			
	Chronic	1,663 (38.1)	1,550 (37.6%)	113 (47.3)			

Data are expressed as mean ± standard deviation or number (%).

df, degrees of freedom; MMSE, Mini-Mental State Examination; GDS, Geriatric Depression Scale.

a, Student’s t-test

b, chi-squared test

Test statistic indicates the t-value in Student’s t-test or the χ^2^-value in the chi-squared test.

### Pain severity and incidence of functional disability

**Fig 1:**
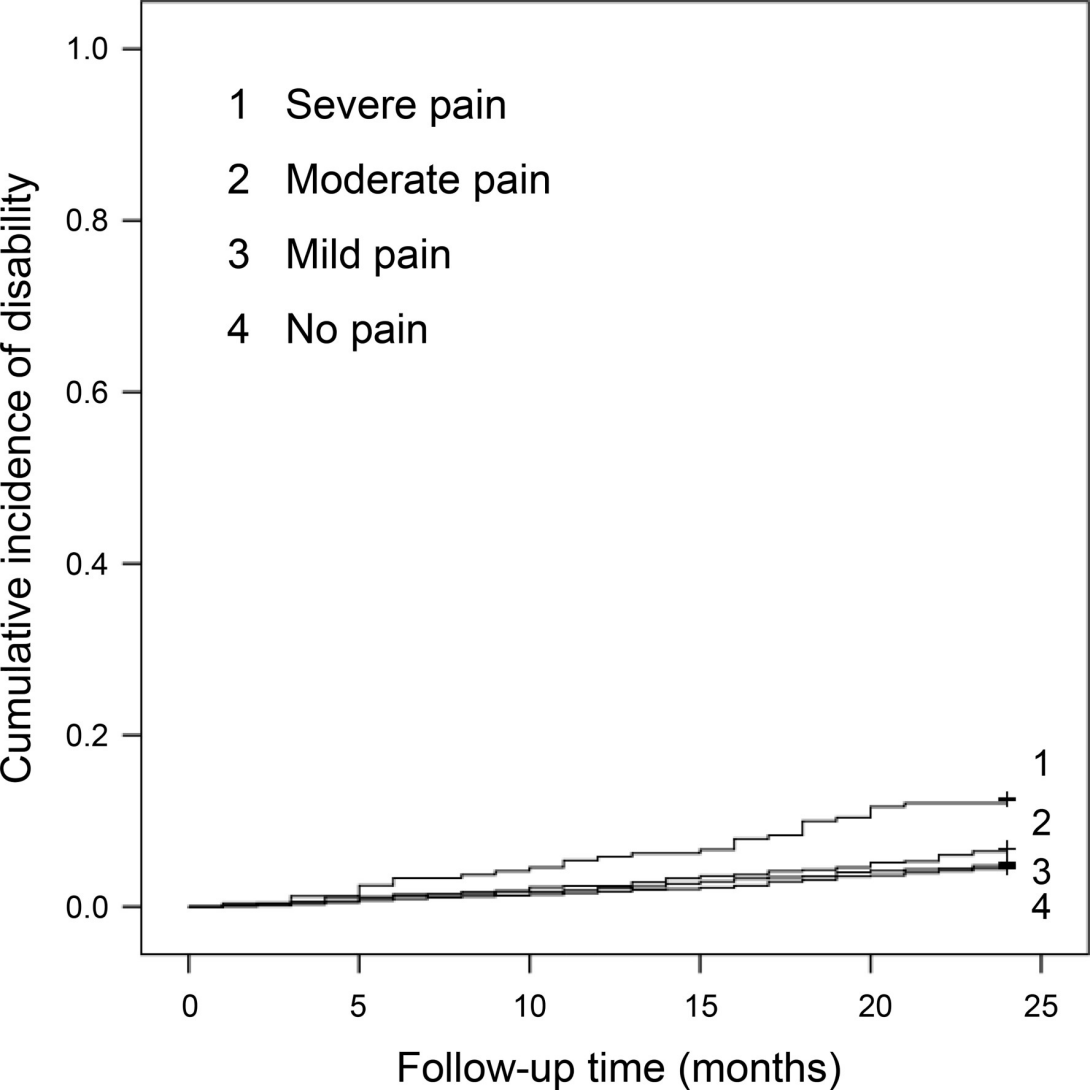
Cumulative incidence of disability according to pain severity.

In the Kaplan-Meier log-rank test, participants with severe pain showed significantly higher risk of disability incidence than those in the other groups (overall *P* < 0.004), and those with moderate pain showed significantly higher risk of disability incidence compared to those without pain (*P* = 0.025). However, mild pain was not associated with disability incidence (*P* = 0.630, Fig 1). Cox regression analysis showed that the hazard ratio (HR) and 95% confidence interval (CI) for disability incidence were 1.08 (95%CI: 0.79–1.49) in mild pain, 1.48 (95%CI: 1.05–2.09) in moderate pain, and 2.84 (95%CI: 1.89–4.27) in severe pain in the crude model and 0.97 (95%CI: 0.70–1.36) in mild pain, 1.00 (95%CI: 0.69–1.47) in moderate pain, and 1.66 (95%CI: 1.05–2.62) in severe pain in the adjusted model (Table 2).

**Table 2:** Hazard ratios (HRs) and 95% confidence intervals (CIs) for disability incidence in the crude and adjusted models for 24 months according to pain severity.

		Crude model				Adjusted model		
		HR	95%CI		*P*	HR	95%CI	*P*
No pain	Reference							
Mild pain	1.08			0.79–1.49	0.630	0.97	0.70–1.36	0.869
Moderate pain	1.48			1.05–2.09	0.026	1.00	0.69–1.47	0.984
Severe pain	2.84			1.89–4.27	<0.001	1.66	1.05–2.62	0.032

The adjusted model was adjusted for age, sex, medical condition (hypertension, diabetes mellitus, heart disease, osteoarthritis, and spinal diseases), polypharmacy (number of medications used ≥ 3/day), slow gait speed (< 1.0 m/s), and depressive symptoms (Geriatric Depression Scale ≥ 5 points)

### Pain duration and incidence of functional disability

**Fig 2:**
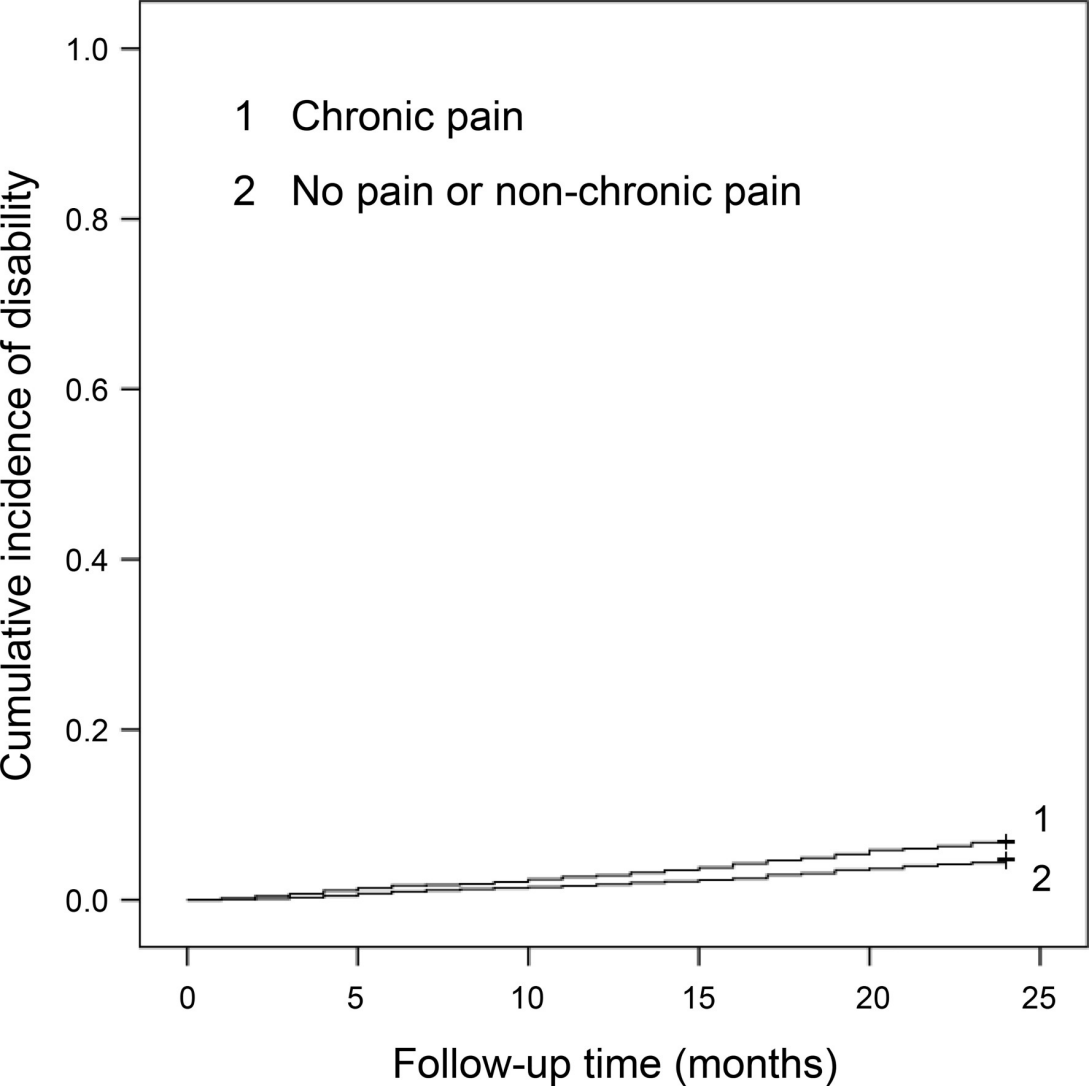
Cumulative incidence of disability according to pain duration.

In the Kaplan-Meier log-rank test, participants with chronic pain showed significantly higher risk of disability incidence than did those without pain or with non-chronic pain (*P* = 0.002, Fig 2). Cox regression analysis showed that the HR and 95% CI for disability incidence were 1.45 (95%CI: 1.15–1.90) in chronic pain in the crude model and 1.02 (95%CI: 0.77–1.35) in the adjusted model (Table 3).

**Table 3:** Hazard ratios (HRs) and 95% confidence intervals (CIs) for disability incidence in the crude and adjusted models for 24 months according to pain duration.

	Crude model			Adjusted model				
	HR	95%CI	*P*	HR		95%CI		*P*
No pain or non-chronic pain	Reference							
Chronic pain	1.45	1.15–1.90	0.003		1.02	0.77–1.35	0.913	

The adjusted model was adjusted for age, sex, medical condition (hypertension, diabetes mellitus, heart disease, osteoarthritis, and spinal diseases), polypharmacy (number of medication use ≥ 3/day), slow gait speed (< 1.0 m/s), and depressive symptoms (Geriatric Depression Scale ≥ 5 points)

## Discussion

In this study, older adults with moderate to severe pain or chronic pain showed a significantly higher risk of functional disability. After adjusting for covariates, functional disability was associated only with severe pain but not with chronic pain.

Previous studies have shown the prevalence of any self-reported pain to be approximately 50–80% among community-dwelling older people [2,3,4,5]. The overall prevalence of pain in this study, 48.7%, was generally consistent with the results of previous studies. The high prevalence of pain suggests an increased necessity for its management. The incidence rate of functional disability during the 24 months was 5.5% among our participants, which was in line with the results of other studies among community-dwelling older adults [23,24].

In terms of pain severity, our results revealed that disability incidence was associated with moderate and severe pain in univariate analyses and with only severe pain in the multivariate analysis after adjusting for covariates. Several previous studies based on the assessment of pain severity (mild/moderate/severe) alone have found that older adults with moderate to severe pain were significantly at risk of disability [2,3]. Our results support these findings, especially the independent association between pain severity and disability. Subjective complaints of pain do not always correspond to physical causes; however, simple questions regarding pain have a certain test-retest reliability (Cohen’s kappa = 0.70 in low back pain and 0.65 in knee pain) [25]. Self-reported severe pain was considered to be a significant risk factor for future functional disability.

In terms of pain duration, disability incidence was associated with chronic pain in the univariate analysis, but not in the multivariate analysis. The temporal relationship between pain duration and functional disability has been rarely reported in previous studies targeting community-dwelling older adults. Our results found that persistent pain affects the incidence of disability, but its impact was relatively small when compared with that of pain severity. Self-report of pain duration required recall of past pain over several months. Therefore, an assessment of pain duration may be more affected by recall bias than that of pain severity; this may result in no independent association with disability.

The major strength of our study was that we examined data from a relatively large cohort including monthly follow-up of functional disability based on the national LTCI system. However, the present study also had several limitations. First, the assessments of pain characteristics were conducted only at baseline; the longitudinal changes in pain and process of medical treatment including pain medication were not considered in our analysis. In addition, we assessed participant pain in terms of worst pain or longest duration by simple interview; this could have affected the detection ability for the relationship between pain characteristics and functional disability. Second, we conducted the health check-up at a community center; the participants were required to be relatively healthy and able to visit the site of the check-up; this might have led to an underestimation of pain prevalence or disability incidence.

## Conclusions

Our findings indicate that moderate to severe pain or chronic pain affects functional disability; in particular, severe pain was independently associated with higher incidence of disability among community-dwelling older adults. Hence, pain management is important in the maintenance of independent living for community-dwelling older adults. Additionally, although subjective complaints of pain do not always correspond to physical causes, simplified questions on pain characteristics may be useful predictors of functional disability in community-dwelling older people.
